# Cue combination and individual differences during weight judgements using familiar and newly learned cues

**DOI:** 10.1038/s41598-025-93947-w

**Published:** 2025-03-29

**Authors:** Olaf Kristiansen, Meike Scheller, Annisha A. Attanayake, Emily A. Bambrough, Marko Nardini

**Affiliations:** Psychology Department, Durham University, Durham, UK

**Keywords:** Psychology, Human behaviour

## Abstract

Human perception is often characterised by efficient combination of sensory signals (cues). In recent studies, people could also improve precision via newly learned cues, with applications to enhance perception in healthy and clinical groups. However, it is unclear whether new cues can enhance manual object interactions. To study how new cues are used for object weight perception, people compared weights of containers. With haptic information plus the familiar visual cue of volume, participants showed precision improvements indicating cue combination. By contrast, a group of participants briefly trained with a novel visual cue to weight (line orientation) did not show improvements expected from combination. We then asked whether prolonged training (12 h) with the novel cue would promote combination, testing for significant precision gains individually in six participants. Half of participants showed combination benefits, but these were not clearly related to training, as some combined cues before training. Using an illusion analogous to the size-weight illusion, we also asked whether the novel cue would become an automatic predictor of weight: two participants were susceptible to the illusion. We conclude that weight perception is susceptible to some enhancement, but subject to training effects and individual differences that are not yet understood.

**Supplementary Information:**

The online version contains supplementary material available at 10.1038/s41598-025-93947-w.

Human perception, across a range of sensory contexts, is characterized by the efficient combination of different sensory signals (cues) that describe the same environmental property. The different cues to a property are individually noisy, but when efficiently combined, they can yield a unified percept less noisy (more precise) than with any of the cues alone. This improvement occurs because averaging independent estimates reduces random noise. Cue combination (CC) benefits have been found across a wide range of tasks, including size perception, depth perception, and spatial navigation^1–3^. For example, in a seminal study by Ernst and Banks^1^, the ability to make fine judgments about which of two objects is taller (that is, discrimination thresholds), improved when both vision and touch were present as compared with the best single cue alone. These improvements were closely aligned with an ideal observer model that takes a reliability-weighted average to reduce random noise. Thus, CC provides a way to enhance the precision of uncertain perceptual judgments, likely playing a role in effective everyday perception and action^4,5^.

While most documented cases of CC have focused on the combination of two familiar cues, sensory signals whose relationship to an environmental property has been learned through a lifetime of experience, CC also provides a means of investigating the adoption of novel cues into perception. For individuals with sensory impairments, such adoption might help them regain some lost abilities through the combination of novel cues (delivered through some sensory substitution device) with whatever diminished native cues remain perceptible, or, for individuals with intact perceptual systems, it could enhance their perceptual abilities.

A few studies have shown some degree of cue combination with novel cues, particularly during visuospatial localization tasks. After brief training, healthy adults showed enhanced precision when using a novel, echo-like audio cue together with visual cues in a VR distance judgment task^6^, indicating that the cues were averaged (combined). Enhanced precision (indicating combination) has also been observed in horizontal localization tasks, involving familiar visual cues combined with novel haptic assistance cues^7^ and a range of novel, arbitrary visual cues such as shape, colour, angle and height^8^. Beyond visuospatial judgments, however, there has been little investigation into combination of novel and familiar cues for enhanced perception^9^. If weight perception could similarly benefit from combination with novel cues, perception could perhaps be enhanced, not only to better know where things are but also what their material properties are, in turn leading to more efficient manual interactions.

Familiar cues like size and material type affect weight perception, as evidenced by weight illusion studies^10^. However, to our knowledge, there has been no demonstration that these cues improve the precision of haptic weight judgments, as happens with other kinds of combination of familiar cues. Regarding novel cues, several studies have found that they can quickly be learned, modulating grip and load forces during lifting tasks^11,12^, but we know of no thorough investigations involving cue combination. One study^13^ examined whether a semi-naturalistic visual cue in a virtual 3D environment would affect weight perception, as participants lifted a virtual object by manipulating a haptic device, leading to the downward visual displacement of the object. The study looked for interference in weight perception caused by the combination of incongruent visual and haptic cues, but found no such effect, possibly due to large differences in the reliability of the individual cues, which would make combination effects difficult to detect^14,15^.

A conventional experimental approach has seemingly not yet been used to determine whether the combination of familiar cues can enhance weight perception, nor whether novel cues have the potential to enhance manual object interactions. In the present study, we aimed first to 1: determine whether familiar cues are efficiently combined in weight perception, and if so, 2: whether novel cues are similarly combined with familiar cues. In Experiment 1a, we measured perceptual precision with either a familiar visual cue (the volume of sand in a jar), a familiar haptic cue (weight felt on the hand), or both together. We found that precision improved when both cues were available compared to either cue alone, suggesting that participants efficiently combined the cues. In Experiment 1b, we introduced a novel, arbitrary visual cue in the form of lines printed on the jar, with their orientation mapped to the object’s weight; participants learned this mapping during a brief training session. Perceptual precision was measured with the novel visual cue, a familiar haptic cue, or both, but was not improved when both were available, suggesting that participants did not combine the cues.

In Experiment 2, we investigated whether novel and familiar cues would be combined after a longer period of training. To that end, we conducted an intensive psychophysical assessment with a small number of participants, with the design and analyses adapted to infer cue combination on an individual basis. We also tested whether the cue would become an automatic predictor of weight by measuring participants’ susceptibility to a weight illusion analogous to the size-weight illusion^10^, in which expectations of an object’s weight can alter the subjective perceived weight.

## Experiment 1a

### Method

#### Preregistration

This experiment’s sample size, included variables, hypotheses, and planned analyses were preregistered at AsPredicted.org (https://aspredicted.org/nd49-9s69.pdf) prior to any data being collected.

#### Overview

**Fig. 1:**
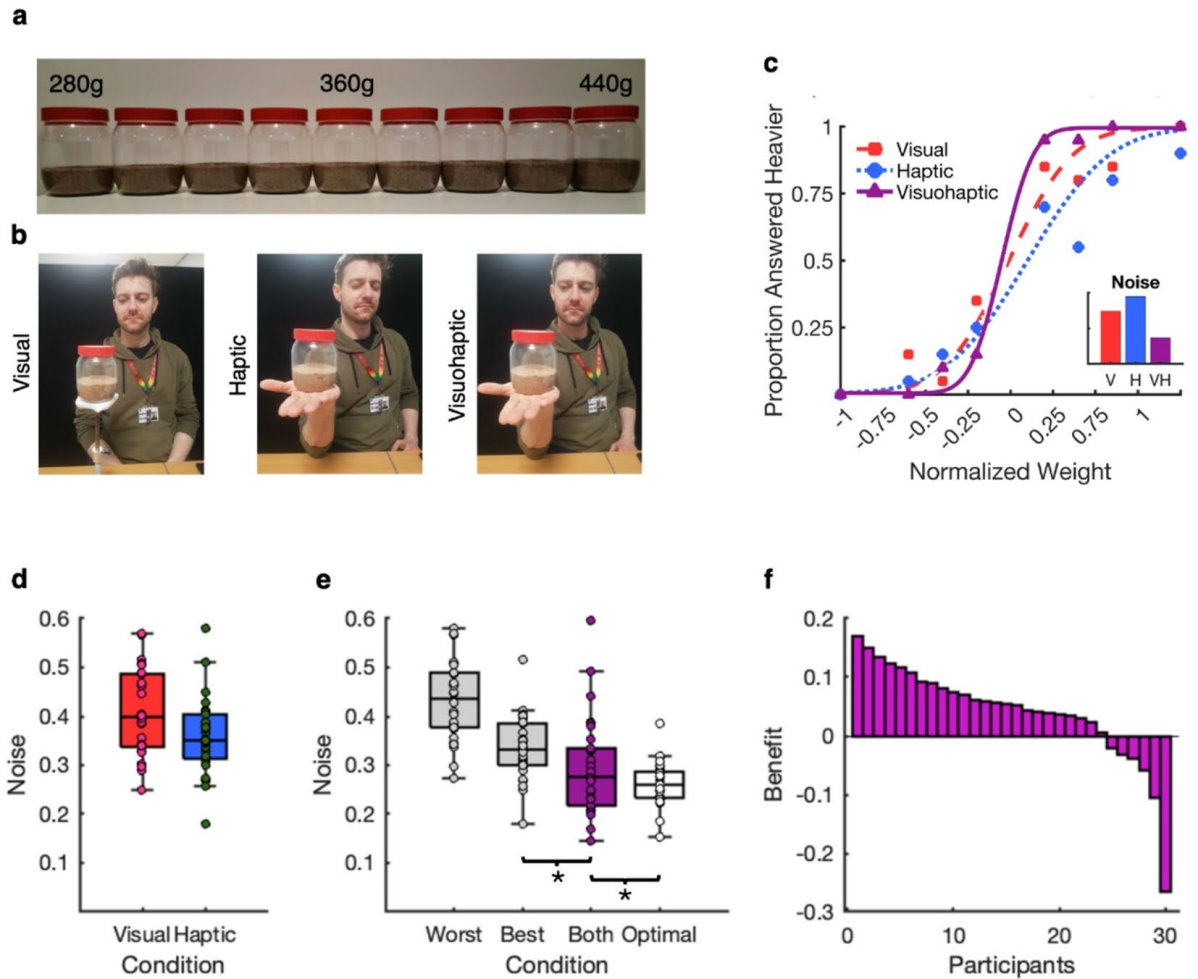
Experiment 1a Method and Results. (**a**) Stimuli. (**b**) Conditions in which participants make 2AFC weight discriminations based on sensory modalities. From left to right: visual, haptic, visuo-haptic. (**c**) Example Participant Psychometric Function. For each participant, psychometric functions are fitted to data across conditions. The example participant’s psychometric function slope is steepest in visuo-haptic condition, reflected in the lower sensory noise compared to the visual and haptic conditions. (**d**) Sensory noise in individual sensory modalities. (**e**) Sensory noise in individual cue conditions by performance, with both cues and theoretically optimal noise. Asterisks indicate a significant difference. (**f**) Combination benefit (noise reduction) compared to participants’ best cue, in order of benefit size.

Participants completed a two-alternative forced choice (2AFC) object weight discrimination task in three cue conditions: visual only (participants viewed the stimuli), haptic only (participants held the stimuli without seeing them), and both (visuo-haptic; participants viewed and held the stimuli). The stimuli were nine transparent jars containing different amounts of sand (Fig. 1a). Based on the cues available in each condition, participants judged on each trial whether the second of two jars was heavier or lighter than the first. For each participant, psychometric functions were fitted for the three conditions and sensory noise parameters, corresponding to the uncertainty of each cue/cue pair were extracted. Non-parametric tests were used to compare sensory noise between conditions. We hypothesized that weight perception judgments in the visuo-haptic condition would be more precise than judgments with participants’ best individual cue.

#### Participants

Thirty participants (24 female; age range: 18–43; mean age: 21.3, SD: 4.8) were recruited through the Durham University Psychology Department’s participant pool and social media. All participants reported having normal or corrected to normal vision and received either participant credits or £10 per hour.

The sample size was based on a power analysis that simulated experiments 1000 times with a range of participant numbers, trial counts, and individual absolute cue noise levels and cue noise ratios. Pilot experiments informed the ranges. All simulated participants were assumed to combine the individual cues. In every simulation, a Wilcoxon Signed-Rank test was used to test whether noise reduction in the visuo-haptic condition could be detected, and we aimed to use participant/trial numbers that achieved at least an 80% probability of detecting simulated combination effects. See Supplementary Information for further details.

#### Ethics

Ethical approval was received from the Durham University Psychology Department Ethics Board, and the experiment was conducted in accordance with all relevant guidelines and regulations. All participants gave written, informed consent prior to taking part in the study.

#### Stimuli

The stimuli were nine transparent jars (Fig. 1a), 115 × 90 mm (height x diameter), containing moldable play sand. Based on piloting with 5 observers, visual and haptic cue levels of comparable reliability were chosen, because well-matched cues provide the most scope for precision improvements from cue combination^14,15^ The jars weighed 280, 312, 328, 344, 360 (reference), 376, 392, 408, and 440 g, and the moldable sand was manipulated so that visual volume varied, with height levels of 34, 36, 36.5, 37, 37.5 (reference), 38, 38.5, 39, and 41 mm measured from the bottom of the jars.

#### Procedure

For each of the three cue conditions, participants completed 160 trials, split into four blocks of 40 trials (12 blocks and 480 trials in total). Two blocks of each condition were completed in each of the two sessions. Each session lasted approximately 90 min. The order of conditions was pseudorandom so that every three consecutive blocks included all three conditions, but the same condition was never repeated for two consecutive blocks. Each block contained five trials with each of the eight comparison jars along with the reference jar, presented in a random order.

In each trial, participants viewed and/or held each jar for around 2.4 s, with a 4.8-second interval between the two presentations. After this, they indicated whether the second jar was heavier or lighter than the first one, using a keyboard. Timing was controlled by a metronome signal that was sent to the experimenter, who placed the jars. Participants could respond as soon as the second jar was presented, but there was no time limit for entering a response.

In the visual condition, a platform was adjusted for each participant so that it matched the height of their hand when held out straight, with their elbow on the table (Fig. 1b), resembling their posture in the other conditions. The top of the platform was covered with foam material to muffle the sound produced when placing the jars. Participants kept their eyes closed while the experimenter placed and removed jars on and off the platform so that the experimenter’s movements would not provide additional information about the jars’ weight. Participants opened and closed their eyes at the experimenter’s instructions to view the jars and then made weight judgments based on the visible volume of sand. In the haptic condition, participants wore glasses that blocked their view of the jar, extended their hands, and had jars placed on their hand by the experimenter. In the visuo-haptic condition, participants also held the jar but followed the experimenter’s instructions to open or close their eyes, as in the visual condition.

#### Analysis

The independent variable for analysis was sensory noise, which corresponds to the inverse of perceptual precision. Sensory noise was estimated for each cue condition separately by fitting a psychometric function of the form:

$$\:P\left(x\:;\:\alpha\:,\:\beta\:,\:\lambda\:\right)=\:\lambda\:+\left(1-2\lambda\:\right)*F(x\:;\:\alpha\:,\:\beta\:)$$

Where *P*(*x*) describes the proportion of the participant judging jar *x* as being heavier than the reference jar. The lapse rate, λ, and guess rate parameters were equated, as appropriate for this kind of task, and were free to vary between 0.01 and 0.20, and fitted individually for every participant in each condition. These represent trials in which participants’ responses were likely not reflective of their actual perception but may have been due to lapses in attention or other extraneous factors. *F* is a cumulative Gaussian distribution function, defined by its location parameter $\:\alpha\:$ and the slope parameter *β*, which the sensory noise s relates to the slope of the function via:

$$\:{\sigma\:=\left(\beta\:*\sqrt{2}\right)}^{-1}$$

Figure 1c shows an example psychometric function. To test for combination benefits, we compared whether sensory noise in participants’ visuo-haptic cue condition was lower than that of the best (lowest noise) individual cue condition (see^15^), using a one-tailed Wilcoxon signed-rank test. Additionally, we tested whether sensory noise in the visuo-haptic condition significantly deviated from optimal predictions based on maximum likelihood estimation (MLE)^1,14^. For each participant, sensory noise from both individual cues *σ*_1_ and *σ*_2_ was used to calculate the optimal sensory noise:

$$\:{\sigma\:}_{b}^{2}\:=\:\frac{{\sigma\:}_{1}^{2}\:{\sigma\:}_{2}^{2}}{{(\sigma\:}_{1}^{2}+{\sigma\:}_{2}^{2})}$$

### Results

Inspection of the individual cue conditions showed that the sensory noise of the visual and haptic cues was well-matched (Fig. 1d). This is confirmed by the average sensory noise ratio of 1.31 between the best cue (visual *n* = 13, haptic *n* = 17) and the worst cue conditions (Fig. 1e). A one-tailed Wilcoxon signed-rank test showed that sensory noise was significantly lower in the visuo-haptic condition (*p* = .002) compared to participants’ best single cue condition, suggesting that participants combined the individual cues. A one-tailed Wilcoxon signed-rank test showed that sensory noise in the visuo-haptic condition was significantly higher (*p* < .001) compared to noise as predicted by MLE, suggesting that participants’ perception was sub-optimal (Fig. 1e). Twenty-four participants showed precision benefits, i.e., a reduction in sensory noise, in the visuo-haptic cue compared to their best individual cue, and six participants showed a precision reduction, i.e., an increase in sensory noise (Fig. 1f).

### Discussion

As hypothesized, weight perception was more precise in the visuo-haptic condition, compared to only the best individual cue. However, perceptual precision in the visuo-haptic condition was still significantly lower than what would be predicted by the MLE model. These results suggest cue combination with familiar cues enhances weight perception, although the combination is not statistically optimal. While examples of optimal cue combination have been found in some contexts (e.g.,^1,2^), there have also been studies showing failures of optimal combination^16^. Six out of the thirty participants performed better with individual cues, which might be due to measurement noise but could also be due to individual variability in cue combination.

Having established that familiar visual and haptic cues to object weight are, on average, combined, we moved on to investigating whether the same would hold true when using a novel visual cue to weight.

## Experiment 1b

### Method

#### Preregistration

This experiment’s sample size, included variables, hypotheses, and planned analyses were preregistered on AsPredicted.org (https://aspredicted.org/hfxc-gbnf.pdf) prior to any data being collected.

#### Overview

**Fig. 2:**
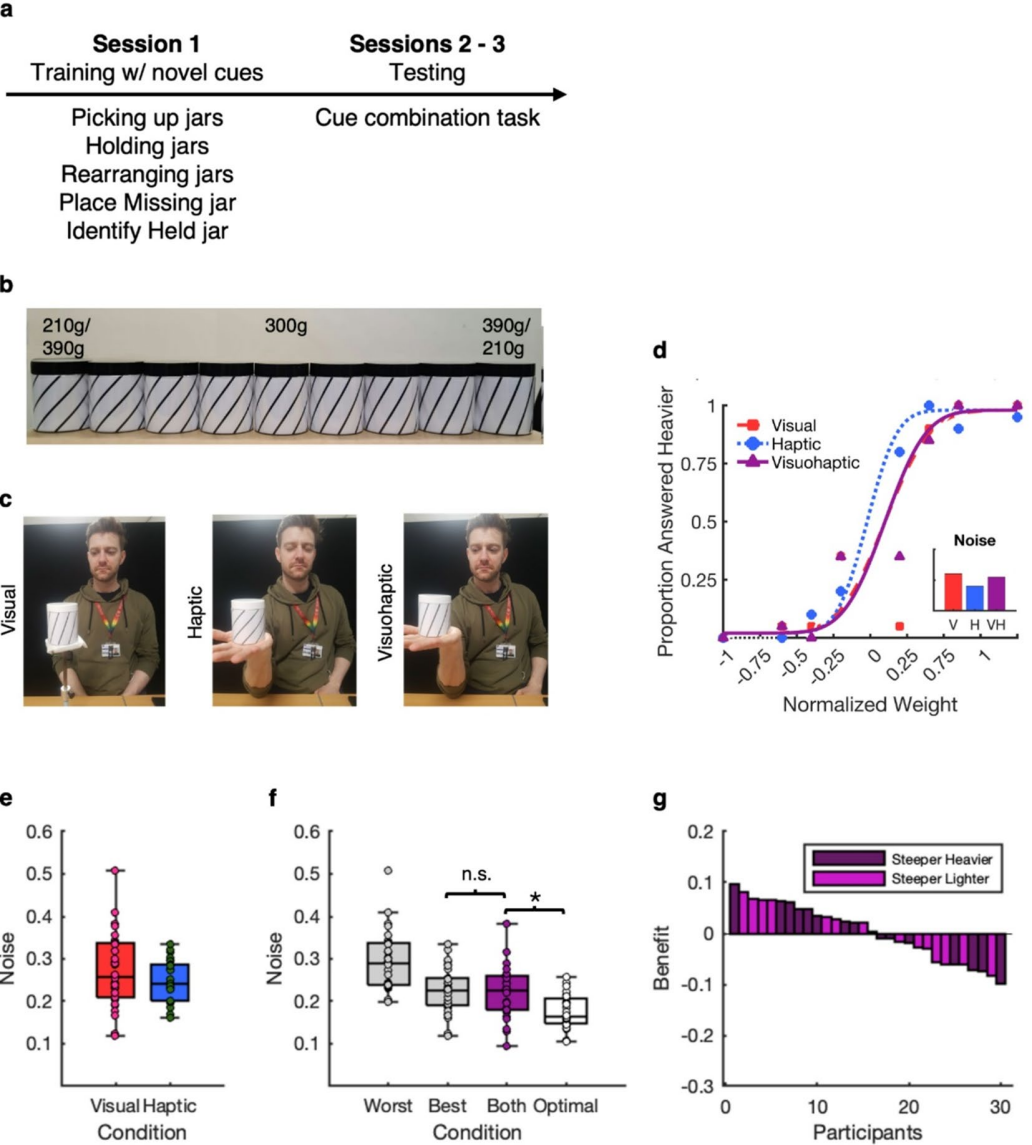
Experiment 1b method and results. (**a**) Experiment timeline. (**b**) Stimuli. (**c**) Conditions in which participants make 2AFC weight discriminations based on sensory modalities. From left to right: Visual, haptic, visuohaptic. (**d**) Example Participant Psychometric Function. For each participant, psychometric functions are fitted to data across conditions. The example participant’s psychometric function slope is steepest in the haptic condition, reflected in the lower sensory noise compared to the visual and visuo-haptic conditions. (**e**) Sensory noise in individual sensory modalities. (**f**) Sensory noise in individual cue conditions by performance, with both cues and theoretically optimal noise. Asterisks indicate a significant difference. (**g**) Combination benefit (noise reduction) compared to participants’ best cue, in order of benefit size. Bar colors indicate the direction of the orientation/weight mapping for individual participants: darker purple marks participants for whom a steeper orientation suggested a heavier weight, and the lighter purple participants for whom a steeper orientation suggested a lighter weight.

As in Experiment 1a, thirty participants completed a 2AFC task spread over two sessions (Fig. 2a), making weight judgments in visual, haptic, and visuo-haptic conditions. A different set of jars was used, each marked with a novel visual cue: lines displayed on the outer surface of the jars, with line angles mapped to jar weight (Fig. 2b). Angles were chosen as a cue on the basis that they were relatively easy to produce in a controlled manner and that participants would likely not have any preexisting association between line angles and weight. However, because crossmodal correspondences exist for a wide range of sensory modalities^17^, some of which could still be unknown, we counterbalanced the direction of mappings across participants. Before starting the 2AFC task, participants completed a series of training tasks to learn the mapping between angles and weight (Fig. 2a). Besides the training, the experiment used the same experimental task and analyses as Experiment 1a, except for minor adaptations to the procedure, described below. As in Experiment 1a, we hypothesized that weight perception judgments with the familiar haptic and the novel visual cues would be more precise than judgments based on either individual cue alone.

#### Participants

Thirty participants (21 female; age range: 18–30; mean age: 22.1, SD: 2.7) were recruited through the Durham University Psychology Department’s participant pool and social media. The sample size was determined using a power analysis similar to that of Experiment 1a. All participants reported having normal or corrected to normal vision and received either participant credits or £10 per hour.

#### Ethics

Ethical approval was received from the Durham University Psychology Department Ethics Board, and the experiment was conducted in accordance with all relevant guidelines and regulations. All participants gave written, informed consent prior to taking part in the study.

#### Stimuli

The stimuli were nine jars measuring 71 × 85 mm (height x diameter; Fig. 2b). As in Experiment 1a, cue levels were selected based on pilot testing to achieve comparable reliability, maximizing the potential for detecting precision benefits from cue combination. The jars weighed 210, 246, 264, 282, 300 (reference), 318, 336, 354, and 390 g. These weights differed from those in Experiment 1a with the goal of reducing sensory noise. Lower sensory noise with individual cues can increase the chances of detecting differences between conditions, despite also potentially reducing the margin for noise reduction in the visuo-haptic condition.

The novel cues were lines of 43°, 48.6°, 51.4°, 54.2°, 57° (reference), 59.8°, 62.6°, 65.4°, and 71°, mapped to the jar weights so that 43° and 71° corresponded to 210–390 g, depending on the direction of mapping (counterbalanced across participants). This angle range was chosen to avoid any prominent orientations, such as 90° or 180°, that might be easily recognized. Angle increments were selected to produce just noticeable differences (JNDs) comparable to the haptic cue and were piloted first on monitors and then printed on paper and inserted into jars, covering the transparent inner surface. The lines were black and printed on white paper, with a width of 3 mm and a 19 mm distance between lines. For the haptic condition, blank white paper was inserted into the jars.

#### Procedure

The 2AFC task was performed as in Experiment 1a (Fig. 2c), the only change being that rather than wearing glasses to block vision, participants opened and closed their eyes in the haptic condition, thus keeping the procedure for the haptic and visuo-haptic condition identical, except using blank jars and jars printed with the novel cue, respectively.

#### Training

To help participants learn the association between weight and the novel visual cue, they completed a series of tasks using the jars with the novel cue. All training tasks were conducted with the jars placed on a wooden stand (60 × 30 × 22 cm: width x length x height), positioned at a similar height to that used during the 2AFC task. Before starting, participants were informed that the tasks would help them learn an association between the visual features of the jars and their weights, but the specific mapping between angles and weight was not explicitly explained. Table 1 provides brief descriptions of the training tasks, with further details in the Supplementary Information.

#### Analysis

As in Experiment 1a, we fitted psychometric functions to estimate sensory noise for each cue condition (see Fig. 2d for example) and tested for combination benefits using a Wilcoxon signed-rank test.

**Table 1:** Brief description of training procedures for experiments 1b and 2 (see supplementary information for further details).

	Experiment 1	Experiment 2
Duration	Single session (~ 60 min)	Eight sessions over two weeks (~ 90 min per session)
Task 1: Lifting Jars	Participants lift jars in various orders.	Same as Experiment 1
Task 2: Holding Jars	Experimenter place jars on participants’ hands in various orders	Same as Experiment 1.
Task 3: Rearranging Jars	Participants rearrange a scrambled set of jars into ascending or descending weight order.	As in Experiment 1, but with additional trials, and difficulty increased in later sessions by swapping certain jars.
Task 4: Placing a Missing Jar	Participants attempt to insert a missing jar in correct position in ascending or descending lineup	Same as Experiment 1
Task 5: Identifying Jars by Touch	Participants hold a jar with eyes closed, then identify it from a lineup. Two iterations: (a) all jars previously held, (b) only one previously held.	Same as Experiment 1
Task 6	N/A (Experiment 2 only)	Participants visually identified a specific jar (e.g., lightest, second-lightest, heaviest, etc.) from a group of 3–5 jars.

### Results

Inspection of the individual cue conditions showed that the sensory noise levels of the visual and haptic cues were well-matched (Fig. 2e), with an average ratio of sensory noise in participants’ best cue condition (visual *n* = 11, haptic *n* = 19) and worst cue condition of 1.36.

A one-tailed Wilcoxon signed-rank test showed no significant difference in sensory noise between the visuo-haptic condition (*p* = .35) and participants’ best single cue condition, suggesting that participants did not combine the individual cues (Fig. 2f). A one-tailed Wilcoxon signed-rank test showed that sensory noise in the visuo-haptic condition was significantly higher (*p* < .001) compared to noise as predicted by MLE, suggesting that participants’ perception was not optimal.

### Discussion

Contrary to our hypothesis, weight perception was not more precise in the visuo-haptic condition, compared to only the best individual cue, and precision in the visuo-haptic condition was significantly lower than what would be predicted by the MLE model. These results suggest that novel cues are not combined after short training to enhance weight perception. Results from Experiment 1a put this result into context: there, using an almost identical method, we found increased precision consistent with combination (albeit sub-optimal) when using a familiar visual cue to weight. Considering the variability of precision benefits in the visuo-haptic condition in Experiment 1a, and 16 out of 30 participants in the current experiment showing benefits (Fig. 2g), there remains the possibility that some participants are combining the cues while others are not.

The brevity of the training may be a limiting factor, as more experience and learning of the mapping between the novel cue and weight may be required to facilitate cue combination. The data collected here also do not allow us to make reliable conclusions about individual participants: it is unclear to what extent individual differences (Fig. 2g) reflect genuine intra-individual variation in cue combination, and to what extent they reflect measurement noise. Thus, we investigated the effects of longer training and possible individual differences in cue combination in a further experiment.

## Experiment 2

### Method

#### Preregistration

This experiment’s sample size, included variables, hypotheses, and planned analyses were preregistered on Open Science Framework (https://osf.io/qt4ph/) prior to any data being collected.

#### Overview

In Experiment 1b, group-level analysis did not reveal evidence of cue combination with a novel weight cue. Participants’ experience with the novel cue was brief, and many participants showed the highest perceptual precision with both cues available, which could reflect true combination benefits for some individuals not detected in a group-level analysis. In the current experiment, we investigated the effect of extended training and used a new method to test participants so that statistical inferences could be drawn on a group level, but also for individual participants. This method requires a much higher trial count per participant than Experiments 1a and 1b, so we adopted a “low N” design, testing six participants. We hypothesized that after 12 h of training, weight judgments in the visuo-haptic condition would be more precise than judgments based on either individual cue alone.

We also investigated whether, after training, the novel cue would become an automatic predictor of weight by testing for an effect akin to the size-weight illusion (SWI)^10^. The SWI occurs when observers hold differently sized objects of identical weight but perceive the smaller object to be heavier than the larger object. A similar effect also occurs with objects that look light or heavy based on their material^18^. While underlying mechanisms are still debated^10,19^, these illusions suggest that expectations about an object’s weight, which are induced by visual aspects of the object, can affect the subjective perception of its weight.

That expectations formed in adulthood can affect subjective weight perception was demonstrated in a study^20^ in which experienced golfers and non-golfers judged the weight of regular and practice golf balls. Practice balls are usually much lighter than regular balls but look nearly identical, with subtle visual differences likely recognizable only by experienced golfers. For the experiment, the regular and practice balls were manipulated to have identical weights. Non-golfers perceived no weight difference between the two types of balls, while experienced golfers, who could differentiate the balls visually and therefore had different expectations of their weight, judged the practice balls to be heavier.

Crucially, weight illusion effects are typically robust to cognitive penetration, and as such, provide a marker for automatic perceptual processing. By testing for a weight illusion, the present study allows us to investigate the extent to which a novel cue might become integrated into weight judgments “automatically”, similar to visual size and material.

In the illusion task, participants lifted two jars of identical weight, one with a novel visual cue suggesting it was heavier and the other suggesting it was lighter. We hypothesized that, after training, participants would perceive the light-cue jar as heavier than the heavy-cue jar. We tested this both before and after training. Additionally, to determine whether participants were susceptible to weight illusions with familiar visual cues, we conducted a weight illusion experiment where the visual cue was a familiar one: the visible volume of sand in the jars.

#### Participants

Six participants (5 female; age range: 19–33; mean age: 22.0, SD: 5.4) were recruited through social media. All participants reported having normal or corrected-to-normal vision and received £12 per hour of participation.

#### Ethics

Ethical approval was received from the Durham University Psychology Department Ethics Board, and the experiment was conducted in accordance with all relevant guidelines and regulations. All participants gave written, informed consent prior to taking part in the study.

#### Stimuli

The jars were like those used in Exp 1b, but to help optimize our adaptive sampling approach to testing and fitting psychometric functions (see below), we increased the range and granularity of the stimulus space that was sampled by including 15 instead of 9 jars. The weights in grams were: 210, 241.5, 255, 264, 273, 282, 291, 300 (reference), 309, 318, 327, 336, 345, 358.5, 390, and angles: 43°, 47.9°, 50°, 51.4°, 52.8°, 54.2°, 55.6°, 57° (reference), 58.4°, 59.8°, 61.2°, 62.6°, 64°, 66.1°, 71°.

**Fig. 3:**
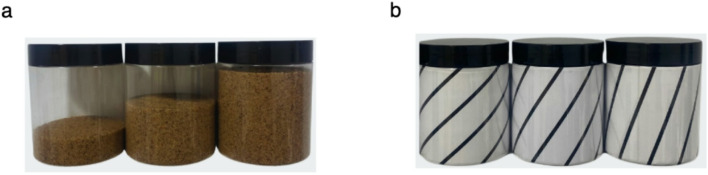
Jars used in weight illusion task. (**a**) Familiar cue (visual volume). (**b**) Novel cue jars (line orientations).

For the familiar cue weight illusion task, the jars used (Fig. 3a) were one containing a small amount of visible sand (20 mm from the bottom), one half-full of sand (42 mm), and one nearly full (64 mm). All jars weighed the same (300 g), which was the weight of the reference jar in the training set and consistent with the natural weight of the middle (half-full) jar. The light-looking jar was made to contain the smallest possible amount of visible sand while still weighing 300 g. To achieve this, ball bearings were attached to the lid’s bottom surface so they were not visible, and inserted at the bottom of the jar and covered with sand. For the heavy-looking jar, tin foil was hidden within the sand to give the appearance of more volume. The jar appeared nearly full, with a visible flat surface left on top, to reduce the likelihood of participants speculating that only the outermost area of the jars was filled with sand, leaving a hollowed center. For the novel cue, the line orientations were 43°, 57°, and 71° (Fig. 3b), with 43° and 71° representing either light or heavy, consistent with individual participants’ training. These orientations represented the lightest and heaviest jar in the training set.

#### Procedure

##### Timeline

**Fig. 4:**
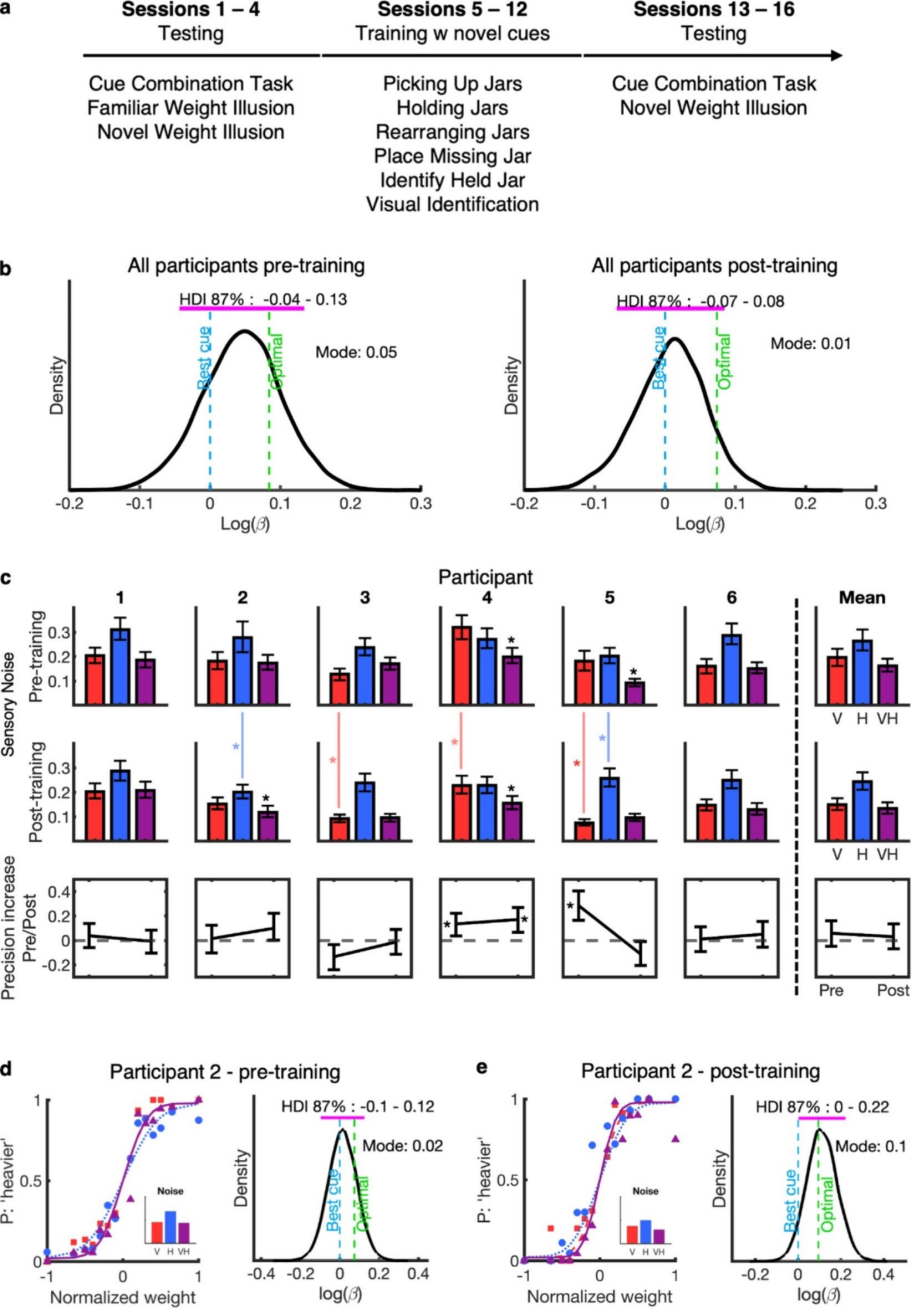
Experiment 2 Results. (**a**) Experiment timeline. (**b**) Group-level HDI plot pre- and post-training, neither of which shows a combination benefit. (**c**) Noise levels (rows 1 and 2) and combination benefits (row 3) pre- and post-training for individual participants. Noise error bars indicate the 87% HDI range. Colored lines between rows 1 and 2 noise bars indicate changes in individual cue precision before and after training. (**d**) Example psychometric function and HDI plot from participant 2 pre-training, showing no combination benefit (left: slope of the VH function is similar to the best single cue; right: the confidence interval for the slope parameter includes the slope of the best single cue). (**e**) Example psychometric function and HDI plot from participant 2, post-training. Left: the slope of the psychometric function is steepest in the VH condition, suggesting a combination benefit. Right: the confidence interval for the reparametrized slope parameter *β* excludes the slope of the best single cue, statistical evidence for a combination benefit.

The experiment took place over 4 weeks (Fig. 4a), with pre-training testing in week 1, training sessions in weeks 2 and 3, and post-training testing in week 4. Each week, participants completed four sessions of either testing or training.

##### 2AFC task

In the main experimental task, as before, participants judged which of two jars was heavier. During the first pre-training testing session, to illustrate the association between jar weight and the novel cue (line orientation), participants were asked to pick up the lightest jar, the middle-weight jar, and the heaviest jar, once in that order and once in reverse.

The 2AFC task was conducted similarly to Experiment 1b but with 344 trials spread across eight blocks for each condition. Unlike Experiments 1a and 1b, which used the method of constant stimuli, this experiment used an adaptive staircase procedure (psi method)^21^ to optimize stimulus selection. Each condition consisted of 43 trials, run within a separate staircase. To optimize the running fit, each staircase function was initialized based on theoretical considerations: each function was based on a cumulative Gaussian function, with the location parameter (alpha) fixed at zero. This decision was made because, in a randomized-order 2AFC task with a reference stimulus, deviations of the location parameter from zero are theoretically negligible and likely attributable only to estimation uncertainty.

The parameter of interest, the slope of the function (beta), which indicates perceptual precision, was allowed to vary within a wide bound (0.06 to 40). The lapse and guess rate parameters were equated, as appropriate for this kind of task, and fixed at 2%. While in Experiment 1, the lapse rate was set to vary, simulations we subsequently ran with analyses like the current one showed that a fixed lapse rate led to less bias in parameters (see also^22^).

##### Weight illusion

The weight illusion task involved both familiar and novel cues. The familiar cue weight illusion was tested before training, while the novel cue weight illusion was tested both before and after training and completion of the 2AFC task (Fig. 4a).

To begin the task, participants first lifted a 300-gram reference jar with no visual cue three times and were informed that this weight represented 100 units. They then lifted three jars, all weighing 300 g, but each with different visual cues suggesting the jars were of light, medium, or heavy weight. Over a total of 75 trials, participants encountered 25 sequences of three jars, which always included a heavy and light cue jar, along with either a medium cue jar (15 trials) or the reference jar (10 trials), in a random order. When the reference jar was presented, participants did not provide a weight estimate; instead, they were reminded that this weight represented 100 units to prevent estimate drift throughout the task. Although only the estimates for the light and heavy cue jars were analyzed, the medium cue jar served to reinforce the weight-to-visual cue mapping and enhance the illusion effect.

On each trial, participants sat with their eyes closed while the experimenter placed the jar on a stand (the same one used in the training sessions). Participants opened their eyes upon hearing a sound played, looked at the jar, and lifted it approximately 5 cm in a smooth motion. After 3.5 s, a second sound played, prompting participants to put the jar back down and report their weight estimate, which the experimenter recorded.

#### Training

Participants completed eight training sessions over two weeks, each lasting approximately 90 min. As in Experiment 1b, participants were informed in the first session that the training aimed to help them learn the association between the lines on the jars and their corresponding weights. Training tasks were similar to Experiment 1b, but with modifications to trial numbers, difficulty, and an additional task. Table 1 contains brief descriptions of the tasks, with additional details in Supplementary Information.

#### Analysis

Psychometric functions were fitted to participants’ 2AFC data using a hierarchical Bayesian model^23^. To assess whether individual participants benefitted from cue combination in their perceptual judgments, we compared the slopes of the best individual cue condition with those of the visuo-haptic condition. The slope of the visuo-haptic condition was reparametrized into a difference parameter, where positive values indicated increased precision compared to the best individual cue condition, and negative values indicated decreased precision. Using Bayesian parameter estimation, we calculated the highest density intervals (HDIs) representing the most likely range for the reparametrized parameter value^23^.

To draw categorical inferences about cue combination, we selected a specific size of the HDI, 87%, at which we can draw inferences about precision enhancements with high power (group level: ~90%, individual level: ~50%) and low alpha error (group level: ~1%, individual level: ~5%), based on simulations (see Supplementary Information). If 0 is not included within this HDI range, and if the mode of the distribution is greater than 0, we conclude that there is a categorical difference between the slopes of the two conditions, indicating higher precision in the visuo-haptic condition.

The location parameter was fixed at 0, as deviations from 0 are not perceptually meaningful in this task due to the randomization of stimulus presentation. The lapse rate was fixed at 2% (see above). The guess rate was set equal to the lapse rate. Given the skewness of the beta distribution, all comparisons were conducted on log-transformed (reparametrized) slope parameters.

When assessing whether bimodal performance is statistically optimal, we tested whether the point estimate of the optimal predictions falls in the HDI range of the reparametrized slope parameter, rather than 0 as a point prediction. The bimodal optimal performance prediction was calculated as:

$$\:{\beta\:}_{VH}^{pred}=\:\sqrt{{\beta\:}_{V}^{2}+{\beta\:}_{V}^{2}}$$

Where *β* is the slope parameter, *V* represents the visual condition, and *H* the haptic condition.

To measure the weight illusion with familiar and novel cues, we calculated the mean weight estimates for the light-cue and heavy-cue jars and used bootstrapping to estimate the 95% confidence interval of the means to determine whether, for each participant and test session, the difference in judged weight for the two jars was statistically different.

## Results

### Cue combination

Psychometric functions were fitted to all individual participants’ data collected before and after training (see Fig. 4a for timeline), and the slope of the visuo-haptic condition was reparametrized into a difference parameter on a group level (Fig. 4b) and individual participant level (Fig. 4c and d-e), with values indicating an increase (positive values) or decrease (negative values) in precision compared to the best individual cue condition).

Before training, the average ratio of sensory noise in participants’ worst (visual worst = 5, haptic worst = 1) to best cue conditions was 1.49, suggesting that the individual cues were reasonably well-matched in reliability. After training, all participants’ best cue was the visual cue, with a mean ratio of 1.87, indicating that the cues were no longer as well-matched. This change was mostly driven by an increase in precision in the novel visual cue condition in 5 participants (see Fig. 4c, top and middle rows).

Assessing the group-level precision parameter of the Bayesian hierarchical model (Fig. 4b) showed that zero was included in the HDI range of the reparametrized slope, both before and after training. However, the average optimal prediction also fell within the HDI. This suggests that variability within the group makes it difficult to distinguish decision strategies—whether participants were optimally combining cues or simply relying on the best sensory cue. Additionally, high precision in the individual cue conditions left limited room for improvement in the visuo-haptic condition, resulting in a small margin within which sensory precision improvements could be established. Notably, since the mode of the group parameter estimate was closer to the best individual cue than to the optimal prediction, this finding supports the notion that the group tended to follow the best individual cue rather than combine the cues. This was observed not only before training but to an even greater extent after training.

At the individual level, pre-training data with the novel cue showed that for two participants (P4 and P5), the reparametrized slope value was positive, with zero falling outside the 87% HDI range. This indicates that perceptual precision was significantly higher in the visuo-haptic condition compared to the best single cue condition (see Fig. 4c, top row). These results suggest that, even before the extended training sessions, P4 and P5 were already effectively combining the familiar and novel cues, having received only basic information about the novel cue and its association with weight. For P4, the optimal sensory precision prediction was within the HDI range, suggesting perception was not different from optimal. For P5, however, the optimal sensory precision prediction was outside the HDI range, indicating that, while P5 did combine the cues, the combination was not optimal. For the remaining four participants, zero fell within the 87% HDI range, suggesting that sensory precision was not categorically higher in the visuo-haptic condition compared to their best individual cue condition (see Supplementary Information for details).

Post-training, P2 and P4 showed categorically higher precision in the visuo-haptic condition (Fig. 4c, middle row). Their optimal precision prediction was within the HDI, suggesting perception was not different from optimal. For all other participants, zero was included in the 87% HDI range, indicating that perception was not categorically more precise when both cues were available.

These differences in precision between the visuo-haptic condition and the best single-cue condition are summarized in the bottom row of Fig. 4c. The plots indicate that participant P4 demonstrated a precision advantage consistent with cue combination both pre- and post-training. Participant P5 exhibited this advantage only pre-training, while P2 showed it only post-training. The remaining participants did not exhibit increased precision from cue combination, neither before nor after training.

It is worth noting that for P5 (see Fig. 4c, top and middle rows), there were categorical differences in precision for the novel visual cue, which increased with the novel visual cue and decreased with the haptic cue. This mismatch in cue reliabilities complicates the measurement of combination benefits—see Discussion. Conversely, for P2, an increase in precision with the haptic cue resulted in better-matched individual cues, making combination benefits easier to detect.

### Weight illusion

**Fig. 5:**
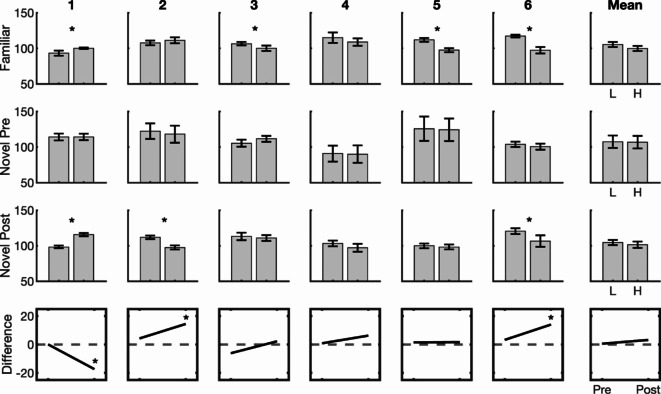
Weight estimates (rows 1–3) in weight-illusion tasks (error bars indicate 95% confidence intervals) and light-cue/heavy-cue estimate differences pre- and post-training (row 4). Asterisks indicate a significant difference.

Weight estimates in the weight illusion tasks using the familiar cue (collected in the first testing session) and the novel cue (collected in the first and final testing sessions, before and after cue training) are shown in Fig. 5. In the familiar weight illusion task, weight estimates for the heavy- and light-looking jars (Fig. 5, top row) differed significantly for three participants (P3, P5, and P6), who estimated the light-cue jar as being heavier than the heavy-cue jar. The non-overlapping confidence intervals indicate that the difference between the two conditions is statistically significant. One participant (P1) perceived the heavy-cue jar as being heavier, experiencing an inverted illusion effect.

Before training with the novel cue (Fig. 5, second row), no participants showed significant differences in weight estimates for the light-cue and heavy-cue jars. After training (Fig. 5, third row), two participants (P2 and P6) perceived the light-cue jar as being significantly heavier (consistent with the classic illusion), while one participant (P1) perceived the heavy-cue jar as being significantly heavier (counter to the classic illusion, but consistent with their judgments with the familiar cue; Fig. 5, top row). The change in illusion magnitude is summarized in the bottom row of Fig. 5. Participants tended to show a positive change (an increase in illusion magnitude), with the post-training illusion reaching significance for two participants. For one participant (P1), however, a significant effect in the opposite direction developed.

### Discussion

Contrary to our hypothesis, after 12 h of training with a novel visual cue, weight perception was generally not more precise when using both visual and haptic cues compared to either individual cue alone. Following training, only two participants, P2 and P4, showed combination benefits, i.e., categorically higher perceptual precision with both cues together when compared to the best single cue (see Fig. 4c). However, P2 was the only participant to show combination benefits only after training, whereas P4 already showed a combination benefit before training, indicating that this benefit was not the result of the lengthy training but was immediately deployed. Furthermore, another participant, P5, who showed a combination benefit before training, did not show the same benefit after training.

A decrease in combination benefits for P5 following training is initially surprising, but an examination of the participant’s noise levels with individual cues may provide an explanation. Before training, the two cues were well-matched in reliability for this participant (Fig. 4c), facilitating the detection of true precision benefits from cue combination^15^. However, following training, this participant showed categorically higher precision with the visual cue compared to pre-training, and lower precision with the haptic cue, leading to a larger cue noise ratio. Larger cue noise ratios reduce the chances of finding true combination effects by decreasing the magnitude of potential precision benefits, which may explain why no precision increase was measured after training. For P4, although the reliability of the visual cue improved, the ratio with the haptic cue remained low, preserving the combination benefit. P2 similarly showed improved precision with the novel cue following training, leading to better-matched individual cues, which may have contributed to the observed combination benefits in the visuo-haptic condition. Across participants, the mean ratio of sensory noise for the two cues was relatively high, which—along with the low sensory noise in the visual condition—may have limited the potential precision benefit of cue combination.

Although our findings show little evidence that 12 h of experience with a novel cue facilitates cue combination, several participants showed significant improvements in precision with the novel cue alone when comparing pre- and post-training performance. While our main interest was in training the use of the new visual cue in a new way (via a novel mapping to weight), it is possible that basic perceptual abilities to make discriminations about the visual stimulus itself also improved in some participants through perceptual learning^24^.

Weight illusions were also tested with familiar and novel cues. With the familiar cue, only three participants were susceptible to the illusion, perceiving the light-looking jar as being heavier (Fig. 5, top row). The absence of an illusion effect for the remaining three participants might be due to the outer edges of objects exerting a stronger predictive influence than the visible volume of objects^25^. In this study, all the stimuli were identical in size and varied only in the visual volume of the sand they contained. Furthermore, compared to typical SWI stimuli, the relationship between weight and volume in sand may be less reliable. Solid materials such as metal or polystyrene foam have a consistent size-to-weight relationship, but sand can be compressed to different degrees, and its density may therefore introduce an additional cue to weight, which undermines the reliability of volume alone as a predictor of weight. The visual differences in sand volume between the light- and heavy-looking jar may also have suggested an unusual volume-weight relationship, casting doubt on its reliability as a weight predictor. These factors together may have contributed to the absence of a reliable illusion effect.

With the novel cue, two participants experienced the illusion after training (none before training; see Fig. 5, rows 2 and 3), suggesting that, for them, the novel cue had become a strong predictor of weight, influencing subjective weight perception similarly to size or material type might^10^. One participant, P1, experienced the inverse illusion with both the familiar and novel cues, perceiving the heavy-cue jar as being heavier. While the traditional SWI effect is sometimes referred to as anti-Bayesian^26^, experiencing the inverse illusion is consistent with a more traditional Bayesian approach to cue combination.

It is unclear to what extent perceptual processes underlying weight illusions and cue combination in weight perception overlap, but it is likely that both rely on learning how the visual and haptic cues covary. Of the two participants who experienced the novel weight illusion (P2 and P6), only P2 showed cue combination benefits, suggesting that the weight illusion does not depend on the efficient combination of the visual and haptic cues (although it is possible that P6 did combine cues, but not to a measurable extent, due to a high cue noise ratio). Conversely, P4 and P5 combined the cues before training but did not experience the illusion, suggesting that the ability to combine the cues does not automatically lead to experiencing the illusion.

## General discussion

As a preliminary step towards investigating whether novel cues can enhance weight perception, Experiment 1a tested and confirmed that weight perception can be enhanced through the combination of two familiar cues, visual volume and haptic feedback. In Experiment 1b, we examined whether a novel cue to weight could similarly be combined with the familiar haptic cue. While approximately half of the participants appeared to gain some precision benefit with both the familiar and novel cues available, these results did not provide evidence that the combination enhanced perception at the group level.

These findings prompted two key questions. First, were the observed increases in precision among individual participants due to genuine improvements in perception, or simply measurement noise? Second, was the brief time afforded to participants for learning the novel cue’s relationship to weight a limiting factor? We explored these questions in Experiment 2 by testing a small sample of participants for combination benefits before and after 12 h of training with a novel cue, using a high trial count and Bayesian hierarchical analysis to investigate perceptual precision on an individual participant basis. Before training, two participants already showed combination benefits, but after training, one of them no longer showed such benefits. The other participant was still showing combination benefits, as was one participant who had not previously done so. These findings suggest that some participants in Experiment 1b may similarly have seen true combination benefits but provide no evidence that the 12 h of experience interacting with the novel cue promoted cue combination. Conversely, when testing whether the cue would induce weight expectations, leading to a weight illusion akin to the SWI, two participants were susceptible to the illusion, suggesting that the training had transformed the novel cue into a strong, automatic predictor of weight.

One of the aims of Experiment 2 was to investigate how experience with the novel cue might affect precision enhancements from cue combination, but we found no evidence of increased benefits. Instead, participants’ perception of the individual novel cue, and the familiar individual cue to a lesser degree, was enhanced after training, leading to the two cues being poorly matched in reliability. This limited the potential for improvement in the task, making combination benefits difficult to detect.

One possible limitation of the study may have arisen from discrepancies in the presentation of the visual cue as in the visuo-haptic condition and the visual condition. In the visual condition, jars were placed on a static platform, whereas in the visuo-haptic condition, they were placed and rested on participants’ hands, where slight movements could introduce some additional noise. If this was the case, comparisons of noise between the visual and visuo-haptic conditions could slightly underestimate the potential combination benefit in the latter.

Nonetheless, we found instances of combination benefits prior to training, which was surprising, but consistent with existing examples of rapid deployment of novel cues^6–8^, in which combination benefits, or task performance improvements, were found with very little, if any, training. These results also lend some support to the possibility that some of the 16 participants in Experiment 1b who showed the highest precision in the visuo-haptic condition, did so due to cue combination, rather than measurement noise.

This raises the question then, why some participants combine while others do not. We conjectured that, in Experiment 1b, limited experience may have been one explanation, and this remains a possibility; the number of participants investigated in Experiment 2 was small, and 12 h of experience is still relatively little. Ernst^9^, who tested for cue fusion with a novel cue before and after one hour of training, found that 3 out of 12 participants showed combination effects before training, while 11 showed combination effects after training, demonstrating that experience with redundant cues can promote their combination. Conversely, as mentioned, this study and others^6–8^ have found improved precision or task performance following minimal experience with the relevant novel cues. This variability may result from individual differences, which could exist on multiple levels. People’s perceptual precision with different cues varies across individuals, and individuals may also differ in their general ability to combine cues. Different types of novel cues may be more easily integrated with familiar cues than others, and if there is a learning process for combining novel and familiar cues, the speed at which this happens could also vary across individuals.

Additionally, if crossmodal correspondences exist between the novel cue and weight (e.g., some evidence suggests that horizontal clothing may make people appear larger^27^), the degree of this correspondence might similarly vary. Exploring the contributions to the development of such individual differences provides avenues for future research. For instance, the role of prior sensory experience, the developmental timescale, and the nature of the novel cue remain unclear. It may be that other novel cues would be more efficiently combined with the familiar haptic cue.

Bayesian causal inference^28,29^ is another framework for understanding differing degrees of cue combination; the framework formulates an ideal observer model in which the observer does or does not integrate cues based on their estimate of the probability that they come from a common source (i.e., “belong together”). Causal inference studies provide a promising avenue for studying failures to integrate new cues, and potentially tracking changes in this with learning. These studies require trials with differing levels of cue conflict and are a potential avenue for future extensions of this research – noting the difficulty that experiencing many conflicting trials (to collect data appropriate for fitting Bayesian causal inference models) can be at odds with learning a consistent new cue mapping.

Overall, the instances of cue combination we observed with minimal training suggest that some individuals can benefit from having a novel cue available for discriminating between weights. Additionally, susceptibility to a weight illusion demonstrated that novel cues can become strong, automatic predictors of weight in some individuals, leading to a change in subjective weight perception. Taken together, our findings suggest potential for enhancing weight perception and manual interactions with objects using augmented, novel cues, but more research is needed to understand the individual factors that determine whether a novel cue can be successfully integrated into native perception.

## Electronic supplementary material

Below is the link to the electronic supplementary material.

Supplementary Material 1

## Data Availability

The data and analysis scripts are publicly available on the Open Science Framework at https://osf.io/qt4ph/.
