# Supplementary material for: Cue combination and individual differences during weight judgements using familiar and newly learned cues
Source: Sci Rep. 2025 Mar 29;15:10881. doi: 10.1038/s41598-025-93947-w (PMC11954982; doi:10.1038/s41598-025-93947-w)
Supplement: Supplementary file 1 — Supplementary Material 1 [file 41598_2025_93947_MOESM1_ESM.docx]

**Cue combination and individual differences during weight judgements using familiar and newly learned cues**

Olaf Kristiansen*^1^, Meike Scheller^1^, Annisha A. Attanayake ^1^, Emily A. Bambrough^1^, Marko Nardini^1^,

*^1^Psychology Department, Durham University, UK*

*Correspondence: [olaf.kristiansen@durham.ac.uk](mailto:olaf.kristiansen@durham.ac.uk) or [olafkrisfo@gmail.com](mailto:olafkrisfo@gmail.com)

**Supplementary Information**

**Supplementary Methods**

**Power Analysis Simulation Details**

**Experiments 1a and 1b**

In pilot tests, we found that the mean sensory noise of participants' best cue was 0.34 for Experiment 1a and 0.24 for Experiment 1b. The mean sensory noise ratio between the best and worst cues was 1.24 and 1.25, respectively. Recognizing that final data may differ from pilot estimates, for both power analyses we conducted 1000 simulations with a range of parameters which we believed could be representative of a larger dataset.

For simulated participants, the best cue sensory noise ranged from 0.15 to 0.6 in Experiment 1a and from 0.1 to 0.4 in Experiment 1b. Cue noise ratios were drawn from a normal distribution centered around 1, 1.5, and 2, with a standard deviation of 0.25. These simulations were run across various participant and trial numbers.

Our power analyses indicated that, with 30 participants and 20 repetitions per weight level (160 trials per condition), a best cue sensory noise of 0.3 (Exp. 1a) or 0.25 (Exp. 1b) and a cue noise ratio of 1 resulted in 98% and 96% power, respectively. With a cue noise ratio of 1.5, power was 92% (Exp. 1a) and 87% (Exp. 1b).

When the best cue noise increased to 0.6 (Exp. 1a) or 0.4 (Exp. 1b), with a cue ratio of 1.5, power was reduced to 79% and 77%, respectively. These results suggest that for a range of plausible parameters, the chosen participant and trial numbers provide an adequate likelihood of detecting a cue combination effect.

**Experiment 2**

We simulated 400 experiments, each with six participants, with best individual cue noise chosen randomly between 0.1 to 0.5 and cue ratios chosen randomly between 1 to 1.5. These values are in line with data from our previous experiment using the same stimuli. We simulated the power and alpha error we obtained (following [1–2]) when including different numbers of trials, and different HDI ranges as inference criteria. Simulation results (Fig. 1) showed that with 325 trials per condition, using a Bayesian hierarchical analysis with an 86–88% HDI cut-off, this results in a statistical power of around 87% and an alpha error of around 1%. Furthermore, individual participant-level models also allowed around 50% power, while keeping the alpha error level around 5%.


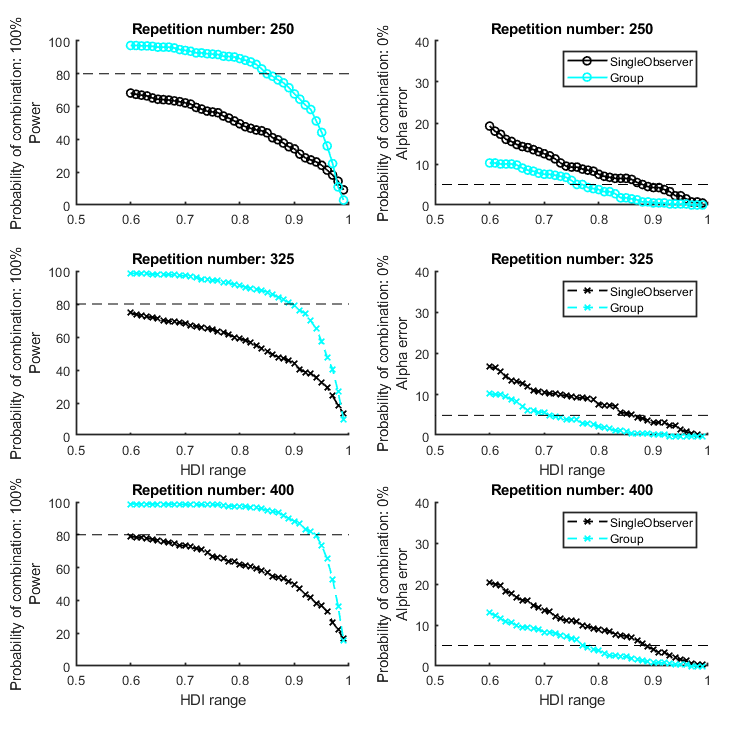


**Supplementary Fig. 1.** Detectability of cue combination effects, assuming combination is present (power) or absent (false positive) for different inference criteria, depending on the width of the HDI range, and trial numbers. Estimates are based on 400 simulations of individual participants (black markers) or groups of six observers (blue markers) using a Bayesian hierarchical model. The criterion for combination was determined as the inclusion of 0 in the re-parameterized slope (hyper-)parameter. Based on these simulations we selected 344 trials and a categorical HDI cut-off of 87%. That means, if 0 were to fall within the 87% HDI interval, we would not assume that participants categorically combined. However, given the probability density distribution of the parameter we could determine where in the credible interval 0 fell, allowing us to directly interpret where there was some (even weak) evidence for cue combination.

We collected data from six adult participants. Participants completed 344 trials in each of the three conditions, both pre- and post-training. According to our simulations, completing 400 trials would yield higher power, but sufficiently high power and low alpha error could be achieved with less trials. Furthermore, increases in task duration risk an increase of lapses, due to participant fatigue and repetitive lifting movements. As 325 trials per condition appeared to lead to sufficient power, providing a lower bound for our estimations, we opted for a small increase in trials to 344 instead of 325, which will produce a small increase in power (i.e. > 87%), when keeping the HDI cut-off for our inference criterion at 87%.

**Novel Cue Training Details**

**Experiment 1b**

**Task 1 – Picking Up Jars** For the first task, all novel cue jars were presented in order of weight on the stand, from left to right, light to heavy, referred to henceforth as jars 1–9. Participants were asked to use their dominant hand to pick up each jar in order, i.e., from jar 1 to jar 9, then picking up all jars in the opposite order, from 9 to 1. The order of the jars on the table was then reversed, and participants lifted jars 9 to 1 from left to right, and 1 to 9, from right to left.

Next, participants again picked up the jars, but now in an order of 1, 3, 2, 4, 3, 5, etc., skipping a jar before moving back a jar. The task was then repeated in the reverse order. Next, the experimenter scrambled the order of the jars, with participants picking up the jars from left to right to left again, before the jars were scrambled once more into another random order and lifted in the same directional sequence.

**Task 2 – Holding Jars** Participants were asked to hold out both hands, palms up, with elbows on the table, as the experimenter placed jars on their hands in a range of sequences. The first sequence was 1 on the left hand and 2 on the right hand, then 2 on the left and 3 on the right, and so on. Next, participants again held two jars in their left and right hands in sequence: 1–3, 2–4, 3–5, and so on, followed by 1–4, 2–5, 3–6, and so on. Finally, the experimenter scrambled the order of the jars and presented them in the scrambled order, e.g., 3–7, 7–6, 6–1, and so on, before scrambling again and presenting the new sequence similarly.

**Task 3 – Rearranging Jars** The experimenter scrambled the order of the jars again, and the participant was asked to rearrange the jars, in order from light (left) to heavy (right). This was repeated a second time, but with the participant rearranging in order from heavy (left) to light (right.) Following this, participants performed the same rearranging task in both directions, but without picking up the jars, thus limiting haptic information and encouraging the use of the visual cues.

**Task 4 – Place Missing Jar** Three jars were placed on the stand, so that two jars were “missing”, e.g., 3–5–7 were presented, with 4 and 6 missing. The experimenter then presented the participant with one of the missing jars, who then had to judge where on the stand it belonged, so that in the above example, they judged whether it belonged between jars 3 and 5 or 5 and 7. Participants were allowed to move the missing jar around to judge in which spot it belonged, but not to pick up the surrounding jars. The experimenter selected jars to keep the task challenging without it getting frustrating. Early trials were easy, and the chosen jars might be further spread apart, e.g., 1–4–7, with the missing jars being 2, 3, 5, or 6. Assuming participants got these correct, later trials got more difficult, ideally reaching a difficulty where participants performed better than expected by chance, but not getting every trial correct.

**Task 5a and 5b – Identify Jar** In task 5a, with eyes closed, participants held out their dominant hand, with palm up and elbow on the table, and the experimenter briefly (~2.5 seconds) placed several jars on their hand sequentially. After this, the jars were placed on the stand in front of the participant (in random order), who then opened their eyes and had to guess, based on the visual cue only, which of the jars they had held last. After giving their answer, participants were told whether they were correct, and if incorrect, which jar was the correct one. This task included 10 trials with two jars, 15 trials with three jars, and 5 trials with four jars. Again, the choice of jars to present was left to the experimenter’s discretion, aiming at a difficulty where performance is above chance, but not perfect.

The final task, 5b, was like the preceding one, but participants held only one jar and then had to pick the held jar out of two, three, or four jars on the stand.

**Experiment 2**

The training setup was similar to that in Experiment 1b, but the stimuli included the expanded range of jars, here referred to as jars 1–15. During the first week of training, participants completed the tasks in the order 1, 2, 3, 4, 5, 6. In the second week, the order was adjusted to 1, 2, 5, 6, 3, 4. Each training session started with participants completing Tasks 1 and 2 (picking up and holding jars), using a subset of the jars, (1, 2, 4, 6, 8, 10, 12, 14, 15), covering the full range of weights without including all 15 jars

In Task 3, participants rearranged the same subset of jars used in Tasks 1 and 2. Both types of rearrangements from Exp. 1b (picking up jars and sliding) were completed three times per session, and on the third time, the difficulty was increased. In the first week of training, the difficulty increase was achieved by replacing the lightest and heaviest jars, jars 1 and 15, with jars 3 and 13, respectively. In the second week, jars 1 and 15 were instead replaced by jars 5 and 11.

Task 4 (placing a missing jar) was performed as in Exp. 1b for five minutes. Fifteen minutes were spent on Tasks 5a and 5b each (identifying held jars). In both tasks, five minutes were spent identifying one out of two, three, and four jars, and the experimenter chose the jars to control difficulty.

Participants completed an additional Task 6, which was not included in Exp. 1b, in which the experimenter placed several jars on the stand and asked participants to identify either the lightest, second-lightest, heaviest, second-heaviest, or middle-weight jar using vision only. This task had three difficulty levels, featuring either three, four, or five jars, with participants spending five minutes on each level.

**Supplementary Results: Individual Participant Result Figures**
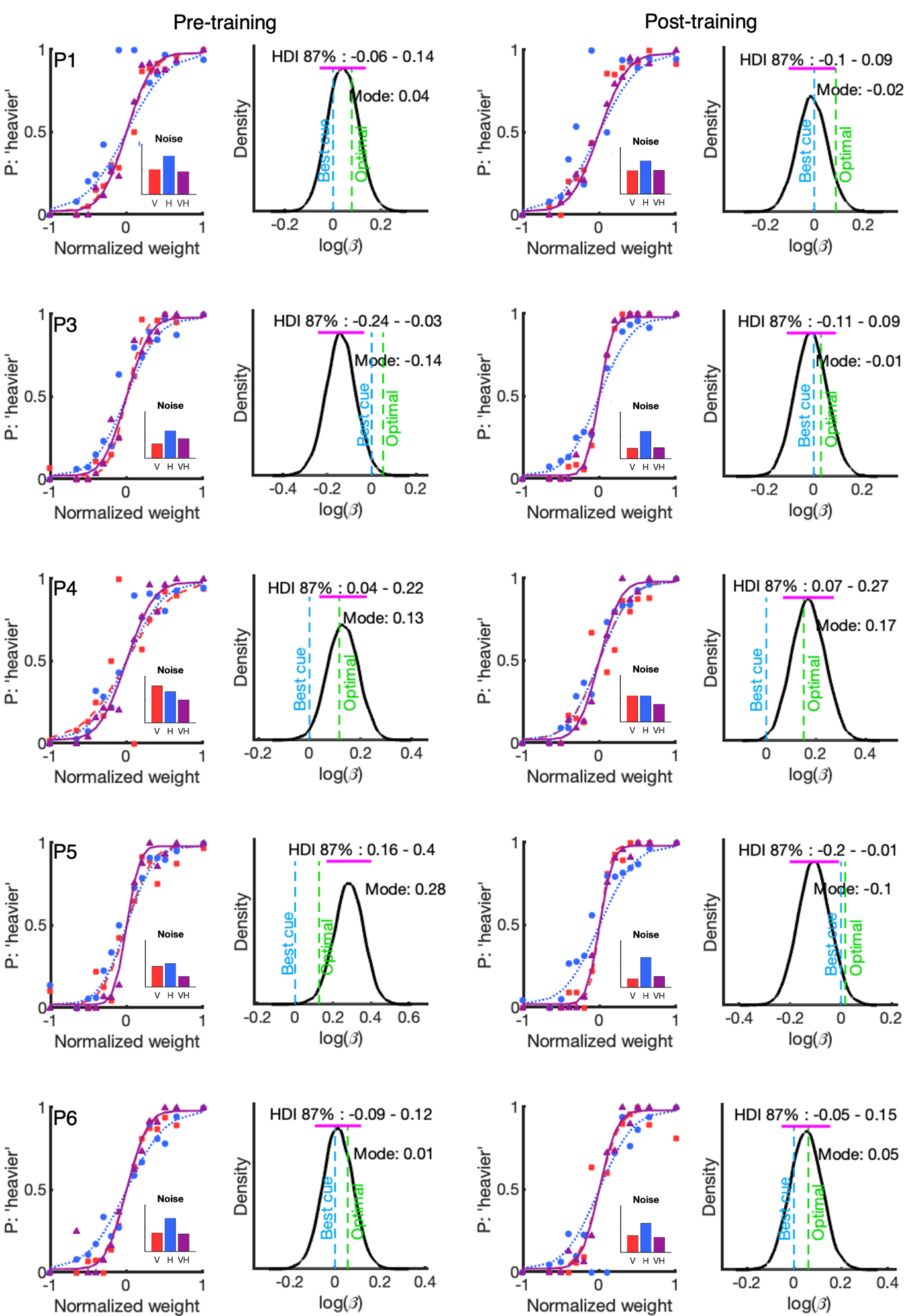


**Supplementary Fig. 2.** Psychometric functions and HDI plots for individual participants (see main article, Fig. 4 d-e for P2),

**Supplementary References**

[1] Scarfe, P. (2022). Experimentally disambiguating models of sensory cue integration. *Journal of Vision*, 22(1), 5-5.

[2] Scheller, M., & Nardini, M. (2024). Correctly establishing evidence for cue combination via gains in sensory precision: Why the choice of comparator matters. *Behavior Research Methods*, 56(4), 2842-2858.
